# Isolation of monomeric photosystem II that retains the subunit PsbS

**DOI:** 10.1007/s11120-013-9914-2

**Published:** 2013-08-24

**Authors:** Patrycja Haniewicz, Daniele De Sanctis, Claudia Büchel, Wolfgang P. Schröder, Maria Cecilia Loi, Thomas Kieselbach, Matthias Bochtler, Dario Piano

**Affiliations:** 1 International Institute of Molecular and Cell Biology, Ul. Ks. Trojdena 4, 02109 Warsaw, Poland; 2 European Synchrotron Radiation Facility, Rue Jules Horowitz 6, 38000 Grenoble, France; 3 Institute of Molecular Biosciences, University of Frankfurt, Max von Laue Straße 9, 60438 Frankfurt am Main, Germany; 4 Umeå Plant Science Center and Institute of Chemistry, Linnaeus väg 10, 90187 Umeå, Sweden; 5 Department of Life and Environmental Sciences, University of Cagliari, V.le S. Ingnazio da Laconi 13, 09123 Cagliari, Italy; 6 Department of Bioinformatics, Institute of Biochemistry and Biophysics, Pawinskiego 5a, 02-106 Warsaw, Poland

**Keywords:** Photosystem II, Photosynthesis, PsbS, Thylakoid membranes, *Nicotiana tabacum*, Oligomeric state

## Abstract

Photosystem II has been purified from a transplastomic strain of *Nicotiana tabacum* according to two different protocols. Using the procedure described in Piano et al. (Photosynth Res 106:221–226, 2010) it was possible to isolate highly active PSII composed of monomers and dimers but depleted in their PsbS protein content. A “milder” procedure than the protocol reported by Fey et al. (Biochim Biophys Acta 1777:1501–1509, 2008) led to almost exclusively monomeric PSII complexes which in part still bind the PsbS protein. This finding might support a role for PSII monomers in higher plants.

## Introduction

Photosystem II (PSII) catalyzes the first light-dependent reaction in oxygenic photosynthesis, the splitting of water molecules into molecular oxygen, protons, and electrons. The proton gradient across the thylakoid membrane then drives the ATP synthesis, while electrons are transferred to plastoquinone and eventually converted to reducing equivalents (Cardona et al. 2012).

PSII seems to occur in both monomeric and dimeric states in vivo. PSII monomers have been associated with the physiological turnover of the dimeric state: typically dimers renew via monomerization and subsequent exchange of the D1 protein, an important polypeptide involved in the process of charge separation and electron transport (Pokorska et al. 2009). Other studies have also suggested that the PSII oligomeric state is dependent on localization. Dimers are reported to occur in thylakoid grana while monomers are predominant in stromal lamellae. Within this distribution, the PSII dimers are considered to be active in oxygen evolution, in contrast to monomers, that are generally less active and heterogeneous (Danielsson et al. 2006).

The PsbS subunit of PSII is considered to be a crucial component in the regulation of the PSII photochemistry, because PsbS mutants are defective in non-photochemical quenching (Li et al. 2000). In contrast to photochemical quenching, which describes the de-excitation of PSII with concomitant electron transport, non-photochemical quenching describes the reduction of PSII fluorescence due to the production of heat (Niyogi et al. 2005). Non-photochemical quenching is controlled by pH in the thylakoid lumen, which has been hypothesized to be sensed by the PsbS protein (Szabó et al. 2005). However, it is not clear how PsbS might mediate the switching of PSII between a fully active state and a protective state of reduced activity induced by the intense light. Prior to the isolation of the PsbS mutant, the xanthophyll cycle was pinpointed as a key player in non-photochemical quenching. Several possible modes of action of the PsbS protein are currently discussed. First, the PsbS protein might influence the xanthophyll cycle (Szabó et al. 2005). Second, the PsbS protein could interact directly with the PSII core (Li et al. 2004; Kiss et al. 2008). Finally, and perhaps more plausibly, the PsbS protein could affect the conformation of the light harvesting complex II (LHCII) (Horton et al. 2005).

Here we report a “milder” extraction of PSII from *Nicotiana* *tabacum,* which resulted in samples constituted mainly of monomeric PSII complexes divided in two populations one of which binds the PsbS protein. This raises the question in which form the functional PSII is organized in vivo in higher plants.

## Results

### Oligomeric state of PSII preparations

**Fig. 1:**
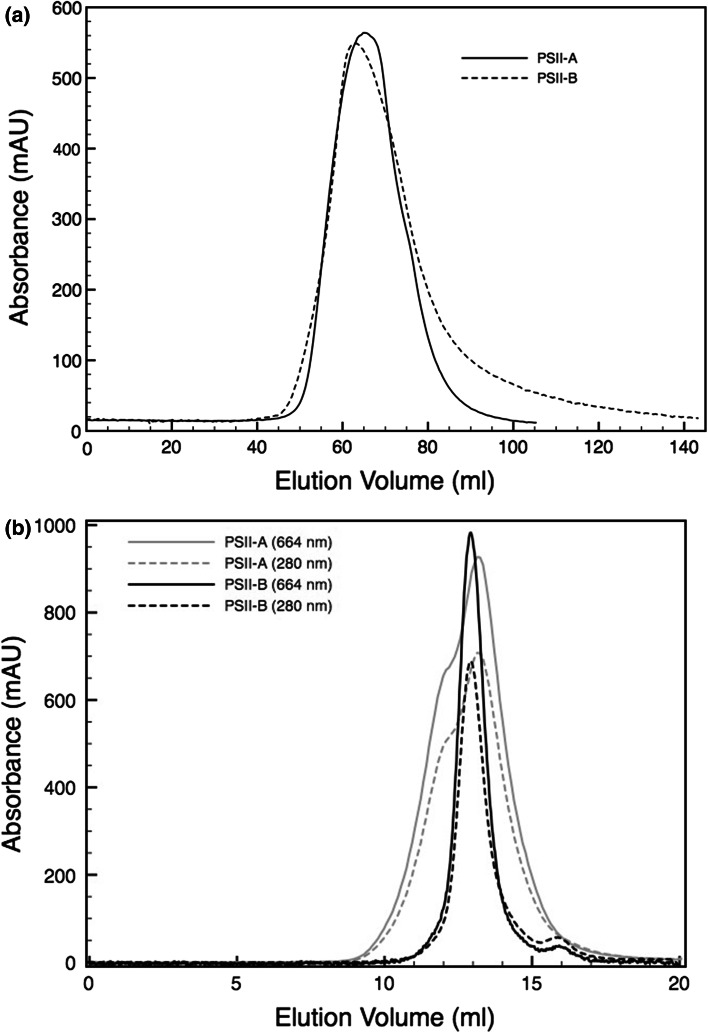

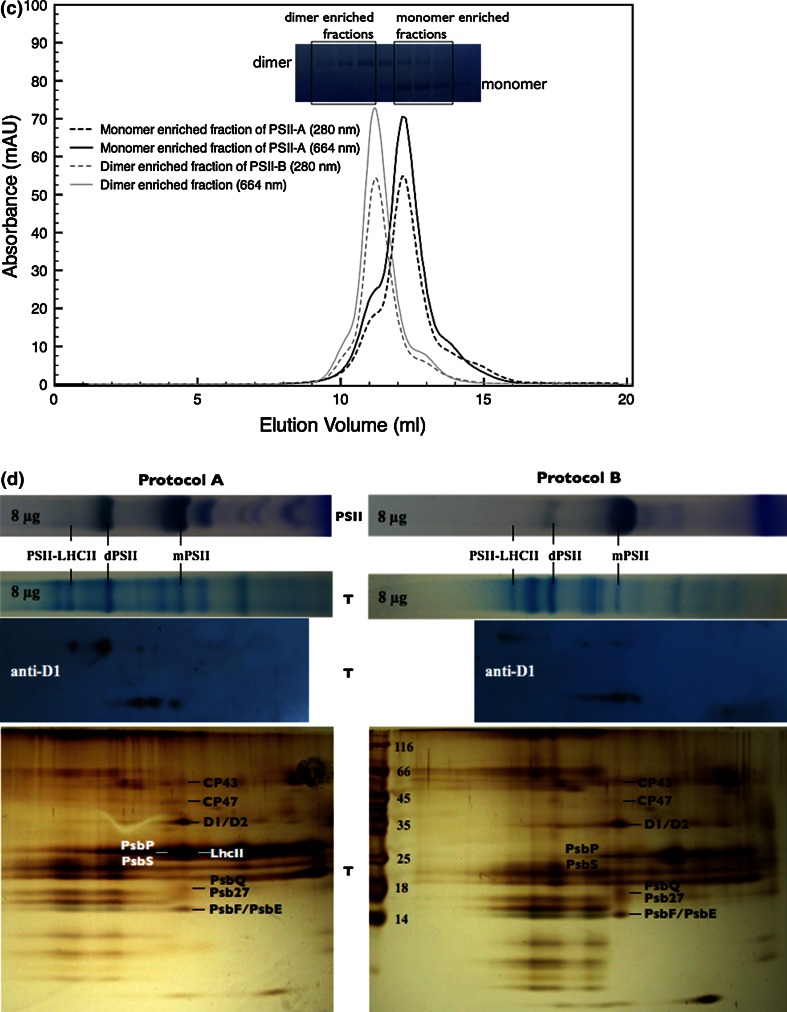
**a** Elution profile recoded at 280 nm of the NiNTA affinity chromatography for the samples prepared according to protocol A (*dashed lines*) and B (*dotted lines*), respectively. **b** Size exclusion chromatography of the PSII preparations. The elution profile of the sample prepared according to the protocol A (PSII-A) is shown in *gray* and the profile of the sample prepared according to the protocol B (PSII-B) is depicted in *black*. Profiles were recorded at 280 nm (*dotted lines*) and 664 nm (*dashed lines*). **c** Size exclusion chromatography recorded at 280 nm (*dotted lines*) and 664 nm (*dashed lines*) of the monomer (*black*) and dimer (*gray*) enriched fractions collected after a previous step of size exclusion chromatography (**b** PSII-A, *gray profile*). Elution fractions compositions of the two pools used for these experiments were analyzed by BN-PAGE (*inset*). The *boxes* in the *inset* indicate the two pools collected for the runs. **d** BN-PAGE of thylakoids (T, 8 μg Chl) solubilized according to protocol A (*on the left*) or protocol B (*on the right*). The *lanes* labeled with PSII show the correspondent PSII samples (8 μg Chl), used as a reference. The *boxes* labeled with anti-D1 represent the western blots for the D1 subunit in the thylakoids after 2nd dimension SDS-PAGE, whereas below the second dimension SDS-PAGES are shown

**Fig. 2:**
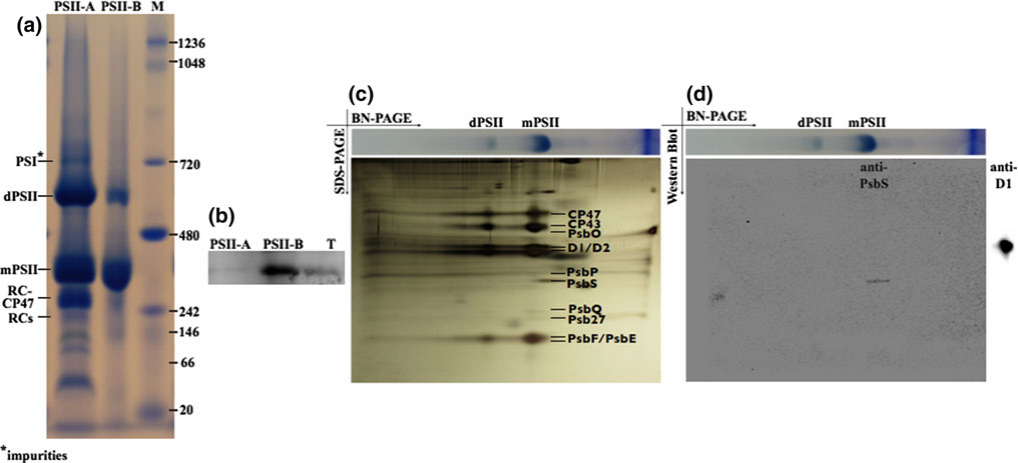
On the *left side* the BN-PAGE of samples obtained with protocol A (*lane PSII-A*) and protocol B (*lane PSII-B*) is shown (**a**), the *lane M* indicates the standard. The associated western blotting reaction using anti-PsbS for the samples PSII-A, PSII-B, and the thylakoids (T) at the level of the PSII monomers is also shown (**b**). Loading was equivalent to 5.1 μg Chl for PSII-A and 3.2 μg Chl for PSII-B. On the *center-right* the second dimension SDS-PAGE obtained after a BN-PAGE of PSII-B as a first dimension is shown (**c**). On the *right* the western blots for anti-PsbS (from the whole gel) and anti-D1 (from the *lane* of monomers) are depicted (**d**)

PSII was isolated from *N. tabacum* plants that had been genetically modified to express the protein subunit PsbE with a hexahistidine tag as described earlier (Fey et al. 2008). Leafs were harvested 5 h before the onset of the light period and PSII complexes were isolated either according to a previously published protocol (Piano et al. 2010, protocol A) or to a new modified “milder” protocol (protocol B), which is based on Fey et al. 2008. In the new method (protocol B) the detergent to chlorophyll ratio was reduced to half and glycerol was included in all buffers. These small alterations had a major effect on the behavior of PSII during purification. In the first chromatography purification step with a Ni–NTA resin, we noted that PSII prepared according to protocol B tended to elute slightly earlier (at lower imidazole concentration) than when using the protocol A suggesting PSII complexes of different subunit composition or alternatively a different monomer to dimer ratio (Fig. 1a). The latter hypothesis was tested by Blue-Native gel electrophoresis (BN-PAGE) confirming that PSII extracted using protocol B migrates mainly in a single band at an apparent molecular mass of 340 kDa representing the monomeric PSII, accompanied by only little amounts of dimers (band migrating at an apparent mass of 680 kDa) (Fig. 2). In contrast, when protocol A was used, several bands were observed, corresponding to the monomer, dimer, and smaller incomplete complexes (Fig. 2). A further step of purification by size exclusion chromatography confirmed the results shown in Fig. 2. In case of PSII extracted with protocol B, a single very sharp peak was observed (Fig. 1b). In contrast, protocol A led to two overlapping peaks, which reflect the presence of different species (Fig. 1b and inset Fig. 1c). The two separated oligomeric forms were found to be very stable over time. Thus, when monomeric or dimeric PSII obtained using protocol A and enriched by size exclusion chromatography were re-injected, they migrated according to the same elution profile, indicating that exchange between monomers and dimers was very slow, if it occurred at all (Fig. 1c) and that the complexes were very stable.

Based on those findings, we used BN-PAGE to analyze the thylakoids solubilized according to protocol A or B. These thylakoids showed different but reproducible separation patterns depending on the solubilization protocol (Fig. 1d). Western blots on second dimension SDS-PAGE helped to identify the main constituents and also to estimate the ratio between PSII monomers and dimers. From those experiments the absence of dimeric PSII in thylakoids prepared according to protocol B was evident by the absence of any anti-D1 signal at the respective mass, whereas when using the harsher protocol A, D1 could be detected for both monomeric and dimeric PSII (Fig. 1d). As observed in other reports, in both cases the D1 signal resulted in two pools of spots equivalent to D1 monomers and D1 aggregates that migrate at almost double of the expected mass (Ishikawa et al. 1999). In order to test whether the results observed were only related to the His-tag present in the transplastomic strain, the same procedure was carried out using wild-type tobacco plants. Those experiments revealed the same solubilization patterns (data not shown).

In order to define whether those results were somehow representative of the composition of the thylakoid membrane, we calculated the yield for both preparations. With the harsher protocol A, all thylakoids (35 mg of total chlorophylls) were solubilized with a final harvest of 0.5 mg of PSII chlorophylls, i.e., a yield of about 1.4 %. On the contrary, with the milder protocol B starting from the same amount of thylakoids only 20 mg of chlorophylls went in solution, i.e., only about 60 % of Chl was recovered. However, from those 20 mg the final amount of PSII chlorophylls harvested was typically 0.4 mg, implying an yield of 2 % of solubilized material or 1.1 % of total Chl. This value is comparable with the recovery observed in protocol A and indicates that the PSII monomeric form is present in roughly the expected amounts judging from total chlorophylls.

### Subunit composition of the two PSII preparations

**Fig. 3:**
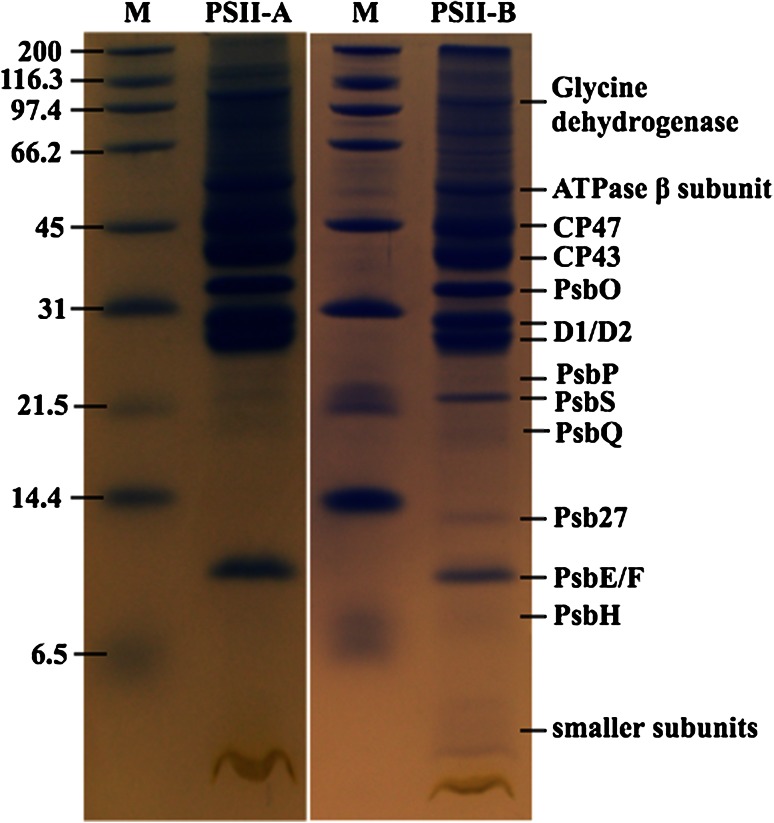
Denaturing SDS-PAGE analysis of PSII preparations according to protocol A (PSII-A) and protocol B (PSII-B). *Lane M* shows the molecular marker. The *labels* for protein bands represent the identifications as found by ESI LC–MS/MS peptide mass finger printing (see Table 1)

The two PSII purified batches were next investigated for their subunit composition by denaturing gel electrophoresis and mass spectrometry. The main PSII core subunits were present in both preparations. However, the samples obtained with protocol B contained the PsbS subunit that was totally absent or only present in trace amounts in samples from protocol A, as shown in Fig. 3.

Further investigation by mass spectrometry (Table 1) shows that protocol A retained four CAB proteins (CAB2, CAB25, CAB26, CAB36). Both preparations contained significant amounts of the subunit CP29 (product of the gene Lhcb4), but none of the major LHCII (polypeptides Lchb1-3). Western Blotting using commercially available polyclonal antibodies confirmed the correct assignment of the different subunits (Table 1). These experiments show that the PsbS protein is present in much higher abundance in B than A samples and that the major LHCII are missing in both preparations. Based on these findings, we will refer to the dimeric fraction obtained from protocol A as PSIId, the monomeric fraction as PSIIm and the monomeric fraction, enriched in PsbS obtained from protocol B as PSIImM (where M stand for Mild). Western blots on the BN-PAGE and on its second dimension SDS-PAGE were performed in order to check whether the presence of PsbS in the PSIImM samples was actually due to the binding, or if it was just the result of a co-migration with PSII monomers. In both cases an anti-PsbS reaction was only observed at the level of PSII monomers, neither in dimers nor as a single PsbS protein. However, when performing BN-PAGE followed by western blotting on thylakoids obtained by protocol B, diffuse signals starting from masses of 360 kDa until 20 kDa were obvious (data not shown). Moreover, we observed also that the single-band obtained from the BN-PAGE on PSIImM samples appeared composite when resolved in second dimension SDS-PAGE (Fig. 2c). This fact suggested that the PSIImM preparation consists of two monomeric PSII populations. Of those two populations the lighter one showed a PsbS band while interestingly the PsbO band was missing (Fig. 2c, d). On the contrary, the PSIImM fraction not able to bind PsbS showed a typical PsbO band (Fig. 2c), suggesting that only one fraction of the total monomers were able to bind PsbS in the PSIImM samples (Fig. 2d). Thus, in the thylakoid membrane PsbS is found in different forms and associations, but especially the results from the second dimension SDS-PAGE provide a strong indication of a specific binding of PsbS to monomeric PSII (Fig. 2).

**Table 1:**
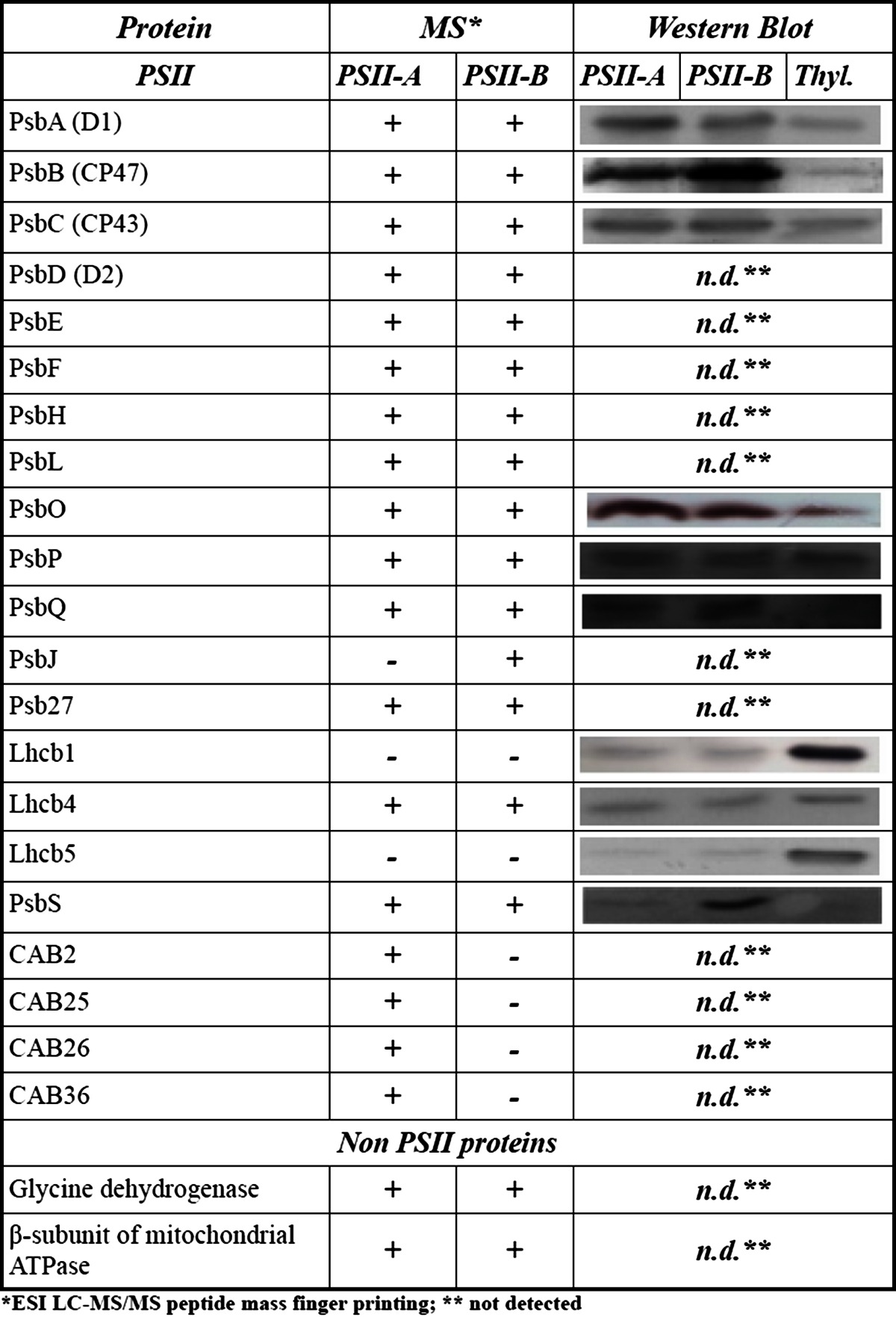
Subunit composition of PSII-A and PSII-B analysed by ESI LC–MS/MS peptide mass finger printing (MS) and western blots in comparison to thylakoids (Thyl). For western blots equal amounts of Chl were load

### Rates of oxygen evolution of the PSII preparations

In order to analyze if the isolated fractions were functionally active we measured the oxygen evolution of the PSIIm, PSIId, and PSIImM samples as well as of both samples obtained after the first purification step (NiNTA elution from protocols A and B). As PSIIm and PSIId are stable and their oligomeric state is not exchanged over time, we could independently determine their activities observing for both high rates of oxygen evolution (Table 2). Surprisingly in the milder extraction, yielding mainly monomeric PSII, only low rates of oxygen evolution (58 μmol O_2_/mg chl h) were observed indicating a much lower activity for the PSIImM sample compared to the PSIIm sample (Table 2).

**Table 2:** Rates of oxygen evolution from isolated His-tagged PSII cores, values are expressed in μmol O_2_/mg Chl h

Preparation	Chromatography step		
	NiNTA	S.E.C.	
	Single pool	1st pool	2nd pool
PSII-A	826 ± 23 (PSIId, PSIIm, RC-CP47, RC)	1100 ± 22 (enriched PSIId)	544 ± 31 (enriched PSIIm)
PSII-B	71 ± 4 (PSIImM, PSIId in traces)	–	58 ± 5 (PSIImM)

Values represent means ± standard deviations of 3 independent measurements from the same preparation

### Spectroscopy of the two PSII preparations

**Fig. 4:**
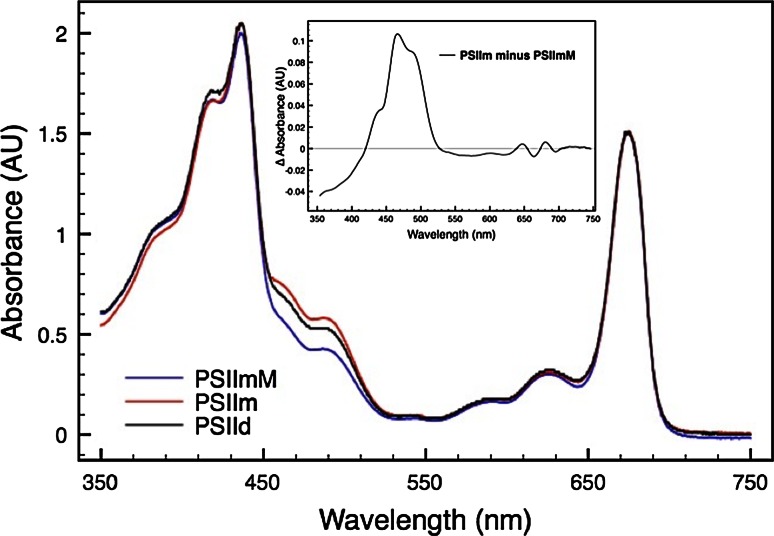
Absorption spectra of the PSIIm (*red line*) and the PSIId (*black line*) from the preparation A and of the PSIImM (*blue line*) from the preparation B. The *inset* shows difference spectrum between monomers (PSIIm minus PSIImM)

Absorption spectra for the PSIIm and PSIId fractions and for the PSIImM sample were recorded in the wavelength range between 370 and 750 nm and normalized to their Q_y_ absorption maximum to facilitate their comparison (Fig. 4). Generally, the three spectra showed a comparable absorption profile regarding the Q_x_ and the Q_y_ regions. However, the intensities differed significantly in the wavelength range between 450 and 520 nm. In this region the absorbance intensity was the lowest for the monomeric PSIImM, followed by PSIId and finally PSIIm. Furthermore, difference spectra between PSIImM and PSIIm feature several characteristic bands. In particular the absorbance at 470 and 490 nm is enhanced in PSIIm, accompanied by minor changes in the Chl b and Chl a Q_y_ region (Fig. 4 inset). These differences indicate the presence of carotenoids, probably associated to some light harvesting proteins in PSIIm, in agreement with the detection of four CAB proteins and the CP29 protein by mass-spectroscopy.

## Discussion

Most PSII preparations described in the literature contain dimers (Boekema et al. 1995; Dekker and Boekema 2005). However, recently a monomeric form in vivo has been reported (Takahashi et al. 2009; Watanabe et al. 2009; Pagliano et al. 2011). Different oligomeric states of PSII have been associated with different locations in thylakoid membranes (Danielsson et al. 2006). Dimers are found mainly in the grana, together with PSII supercomplexes that consist of dimers associated with antenna proteins (see Fig. 5; Table 4 in Danielsson et al. 2006). PSII monomers are located mainly in the margins of the grana, in the stroma lamellae and in the distal region of the stroma lamellae, the so-called Y100 region. Immunogold labeling experiments performed on maize thylakoids using antibodies against PsbS have shown that PsbS tends to be associated to stroma lamellae in leaves exposed to an intermediate or intense light regime (Teardo et al. 2007) similar to the one used in this work. However, some reports have also shown PsbS strongly associated to the grana (Kiss et al. 2008; Horton et al. 2008; Kereïche et al. 2010) suggesting an ubiquitous localization of this protein in thylakoid membranes.

We suspect that the “milder” PSII purification protocol B reported here solubilizes only monomeric PSII present in the stroma, while the “harsher” protocol solubilizes also PSII from the internal grana cores. As shown in Fig. 1d, the thylakoids solubilized following the two different protocols present different patterns. In particular from western blots analysis using anti-D1 the milder protocol seems to contain only PSII monomers and some weak signal at higher molecular weight due to traces of PSII-LHCII supercomplexes; on the contrary in the harsher protocol the signals are most pronounced at the level of the PSII dimers. According to this interpretation, PSIId could be considered of grana origin, whereas PSIIm would represent an enrichment of PSII of lamellar origin. The presence and (near) absence of PsbS in our two samples would then reflect the physiological association with PSII, i.e., PsbS would be preferentially attached to stromal PSII (PSIImM). This is still in line with the observations by Fey et al. (2008), where PsbS was also reported to be present in PSII cores. In those preparations probably all PSII complexes were isolated, and as in our PSII-A the PsbS content was relatively low. The composite constitution of the PSIImM samples (Fig. 2c) is due to the presence of two sub-populations of monomeric PSII in which one of them contains PsbS and lacks PsbO. As PsbO is important for the stabilization of the oxygen evolving center (Yi et al. 2005), this fraction can be expected to be non-functional on the one hand and highly sensitive to photo-damage on the other hand. Hence, the presence of PsbS in the PsbO deficient population is mechanistically reasonable. This sub-population of PSII monomers is probably similar to the lamellar PsbO-deficient PSII particles observed by Bassi et al. (1995) and to the inactive monomeric PSII present in the Y-100 domain reported by Danielsson et al. (2006). Finally, the other sub-population of PSIImM that contains PsbO, but lacks PsbS could originate from the stroma-lamellae domain. This assignment would agree with previous observations of a partially active PSII monomer in this region of the membranes (Danielsson et al. 2006).

## Materials and methods

### Growth and cultivation of tobacco plants

The transplastomic plants of *N.* *tabacum*, that carry a hexa-histidine tag sequence at the 5′ end of the gene coding for the PsbE subunit, were described by Fey et al. (2008). The plants were kept at a constant temperature of 25 °C at 50 % relative humidity and grown for 10–12 weeks under a light regime of 12 h/day, with a light intensity of 150–200 μmol photons/(s m^2^).

### Thylakoid preparation

Thylakoid membranes were purified as reported previously by Fey et al. (2008) with only minimal modifications in the solubilization step. In brief, thylakoids were resuspended in 20 mM MES–NaOH, pH 6.5; 100 mM NaCl; 5 mM MgCl_2_; 10 mM NaHCO_3_; 12.5 % (v/v) glycerol prior solubilization. PSIImM core complexes were obtained from thylakoids membranes solubilized for 5′ at 4 °C at a final chlorophyll concentration of 3 mg/ml (protocol B). The PSII core complex lacking of PsbS (protocol A) was prepared starting from thylakoids membranes solubilized for 15′ at 4 °C at a final concentration of 1 mg/ml chlorophyll. In both cases solubilization was carried out using 20 mM β-dodecylmaltoside (β-DDM).

### PSII core complex purification by affinity chromatography

Photosystem II samples were prepared using Ni affinity chromatography. PSII isolated following the protocol A was prepared according to Piano et al. (2010); PSII isolated following the protocol B was prepared according to Fey et al. (2008) with minor changes. In brief, for the protocol A the washing buffer was free of glycerol (20 mM MES–NaOH, pH 6.5; 100 mM NaCl; 10 mM NaHCO_3_; 15 mM imidazole; 1 M betaine). For protocol B the washing buffer consisted of 20 mM MES–NaOH, pH 6.5, 100 mM NaCl, 10 mM NaHCO_3_, 15 mM imidazole, 1 M betaine, 12.5 % (v/v) glycerol. In both cases PSII cores were then eluted using 40 mM MES–NaOH, pH 6.5; 20 mM NaCl; 5 mM MgCl_2_; 1 mM CaCl_2_; 10 mM NaHCO_3_; 300 mM imidazole; 1 M betaine. In both preparations the washing and the elution buffers contained 0.02 % instead of 0.03 % (w/v) β-DDM. The volumes of washing were increased to 12 CV.

### Size exclusion chromatography

Both preparations were concentrated using Vivaspin 20 ultrafiltration membranes with 100 kDa cutoff until a final volume of 500 μl. The protein sample was loaded on a gel filtration column (Superose 6 10/300 GL, GE Healthcare) equilibrated with gel filtration buffer (40 mM MES–NaOH, pH 6.5; 20 mM NaCl; 5 mM MgCl_2_; 1 mM CaCl_2_; 10 mM NaHCO_3_; 0.02 % (w/v) β-DDM). The main peaks were pooled and concentrated by ultrafiltration (Vivaspin 20, 100 kDa cutoff) to a volume of 200 μl and when necessary re-injected for a second separation.

### Absorption spectroscopy and chlorophyll determination

Thylakoid protein content was measured referring to the Chl a and Chl b concentrations. The analysis was done photometrically in 80 % (v/v) acetone using a Pharmacia Biotech Ultrospec 4000 spectrophotometer and Chl concentrations were calculated according to Porra et al. (1989). Absorption spectra were recorded at room temperature in the range of 370–750 nm with an optical path length of 1 cm and a band-pass of 2 nm.

### Polyacrylamide gel electrophoresis and western blots

For denaturing SDS PAGE, 10 % (w/v) separating polyacrylamide/urea gels with 4 % (w/v) stacking gels were used (Schägger and Jagow 1987). Samples were denatured with Rotiload (Roth) at room temperature before loading, and after the electrophoretic separation the gels were stained with Coomassie brilliant blue G250. Blue native gel electrophoresis was carried out using 3–12 % (w/v) continuous gradient gels according to Schägger and Jagow 1991. PSII complexes at 0.2 mg Chl/ml were mixed with 0.25 volumes of Coomassie Blue Solution (5 % (v/v) serva Blue G, 750 mM aminocaproic acid, 35 % (w/v) sucrose). Electrophoresis was carried out at 205 V for 5 h at 4 °C. For 2D separation, the strips from the BN-PAGE were excised and denaturated with Rotiload (Roth) at room temperature for 20 min. After denaturation the strips were placed on the top of a denaturing SDS-PAGE as described above and sealed with Agarose 0.5 % in cathode buffer. For Western blots, gels were first equilibrated in cathode buffer (25 mM Tris/HCl, pH 9.4; 40 mM glycine; 10 % (v/v) methanol). For transfer of the proteins onto a PVDF membrane, filter papers soaked in two different anode buffers (0.3 M Tris/HCl, pH 10.4; 10 % (v/v) methanol and 25 mM Tris/HCl, pH 10.4; 10 % (v/v) methanol) and in cathode buffer were used. Transfer was carried out for 30–60 min, at a current of 1.5 mA/cm^2^. The membranes were treated with the antisera (purchased from Agrisera, Sweden) solutions, the resulting bands visualized by ECL (Amersham) and signals were recorded on X-ray film (Kodak). Stripping of the antibodies in order to probe one blot with different antibodies was carried out as recommended by the manufacturer of the ECL kit.

### Mass spectroscopy

The in-gel digested samples were analyzed by ESI LC–MS/MS using an HCT ultra ETD II iontrap instrument (Bruker) linked to an Easy nano LC system (Proxeon). Processing, deconvolution, and compound detection for the LC–MS/MS datasets were performed using the Data Analysis software (4.0 SP4, Bruker). Database searches using the peak lists files of the processed datasets were performed using an in-house license of the Mascot search engine (Matrix science) and the current version of the Uniprot database (2012_01). The search parameters permitted a mass error of 0.3 Da for both the MS and the MS/MS mode and variable modifications of methionine by oxidation, of cysteine by propionamide derivation and N-terminal acetylation.

### Oxygen evolution

Oxygen evolution was assessed with a Clark-type electrode (Hansatech, England) at 20 °C in gel filtration buffer with 1 mM 2,6-dichloro-*p*-benzoquinone, and 1 mM ferricyanide as electron acceptors in the reaction mixture.
